# Comparing Neuroplasticity Changes Between High and Low Frequency Gait Training in Subacute Stroke: Protocol for a Randomized, Single-Blinded, Controlled Study

**DOI:** 10.2196/27935

**Published:** 2022-01-28

**Authors:** Fatimah Ahmedy, Natiara Mohamad Hashim, Herwansyah Lago, Leonard Protasius Plijoly, Ismail Ahmedy, Mohd Yamani Idna Idris, Abdullah Gani, Syahiskandar Sybil Shah, Yuen Kang Chia

**Affiliations:** 1 Faculty of Medicine and Health Sciences Universiti Malaysia Sabah Kota Kinabalu Malaysia; 2 Faculty of Medicine Universiti Teknologi MARA Sungai Buloh Malaysia; 3 Faculty of Engineering Universiti Malaysia Sabah Kota Kinabalu Malaysia; 4 Faculty of Computer Science and Information Technology University of Malaya Kuala Lumpur Malaysia; 5 Faculty of Computing and Informatics Universiti Malaysia Sabah Kota Kinabalu Malaysia; 6 Department of Rehabilitation Medicine Queen Elizabeth Hospital Kota Kinabalu Malaysia; 7 Department of Internal Medicine Queen Elizabeth Hospital Kota Kinabalu Malaysia

**Keywords:** neuroplasticity, gait training, stroke rehabilitation, electroencephalogram signals

## Abstract

**Background:**

Walking recovery post stroke can be slow and incomplete. Determining effective stroke rehabilitation frequency requires the assessment of neuroplasticity changes. Neurobiological signals from electroencephalogram (EEG) can measure neuroplasticity through incremental changes of these signals after rehabilitation. However, changes seen with a different frequency of rehabilitation require further investigation. It is hypothesized that the association between the incremental changes from EEG signals and the improved functional outcome measure scores are greater in higher rehabilitation frequency, implying enhanced neuroplasticity changes.

**Objective:**

The purpose of this study is to identify the changes in the neurobiological signals from EEG, to associate these with functional outcome measures scores, and to compare their associations in different therapy frequency for gait rehabilitation among subacute stroke individuals.

**Methods:**

A randomized, single-blinded, controlled study among patients with subacute stroke will be conducted with two groups: an intervention group (IG) and a control group (CG). Each participant in the IG and CG will receive therapy sessions three times a week (high frequency) and once a week (low frequency), respectively, for a total of 12 consecutive weeks. Each session will last for an hour with strengthening, balance, and gait training. The main variables to be assessed are the 6-Minute Walk Test (6MWT), Motor Assessment Scale (MAS), Berg Balance Scale (BBS), Modified Barthel Index (MBI), and quantitative EEG indices in the form of delta to alpha ratio (DAR) and delta-plus-theta to alpha-plus-beta ratio (DTABR). These will be measured at preintervention (R0) and postintervention (R1). Key analyses are to determine the changes in the 6MWT, MAS, BBS, MBI, DAR, and DTABR at R0 and R1 for the CG and IG. The changes in the DAR and DTABR will be analyzed for association with the changes in the 6MWT, MAS, BBS, and MBI to measure neuroplasticity changes for both the CG and IG.

**Results:**

We have recruited 18 participants so far. We expect to publish our results in early 2023.

**Conclusions:**

These associations are expected to be positive in both groups, with a higher correlation in the IG compared to the CG, reflecting enhanced neuroplasticity changes and objective evaluation on the dose-response relationship.

**International Registered Report Identifier (IRRID):**

DERR1-10.2196/27935

## Introduction

Stroke is one of the most common causes of death and acquired disability worldwide, affecting motor function, speech, swallowing, vision, sensation, and cognition, and poststroke recovery can be slow and incomplete [1,2]. Stroke has been reported by Malaysia’s Ministry of Health to be the third leading cause of mortality and morbidity [3]. The rehabilitation process should begin within the first few days after stroke to maximize neurological and functional recovery to achieve the highest possible level of independence. However, rehabilitation therapy for stroke is long term and time-consuming, and can be expensive with the use of advanced equipment or gadgets [4,5]. Hence, determining effective and tailored stroke rehabilitation therapy within limited resources is a matter of priority [6]. Rapid and accurate decision-making is critical to stroke rehabilitation care, for which several factors have proven to affect the stroke outcomes, including therapy frequency, intensity, and task-specific training [7].

Motor learning through stroke rehabilitation is proven to promote cortical reorganization and neuronal synaptogenesis that form the basis of the neuroplasticity concept [8]. Neuroplasticity is the ability for the brain to repair and reorganize after acquiring an injury such as stroke and is primarily affected by three main factors: therapy frequency, intensity, and task-specific training [9-11]. At present, choosing the best rehabilitation therapy for stroke patients is, in part, a trial-and-error process that can take weeks. However, recovery capacity after stroke declines overtime in which maximum recovery is demonstrated within the first 6 months after stroke [12]. It is imperative to choose an effective rehabilitation regime for promoting neuroplasticity within the recovery period. For the majority of clinical settings in lower-income regions, rehabilitation medicine experts assess and decide the best rehabilitation therapy for poststroke patients. Decisions are made based on subjective assessments that may result in contradicting patients’, families’, and relatives’ expectations, and potentially inappropriate advice, treatment, or discharge. Hence, an objective assessment is needed to demonstrate and prove neuroplasticity changes with stroke rehabilitation.

One of the ways such changes can be identified is through a neurophysiological study, which is captured as neurobiological signals. These specific signals can be sensed several ways, but established studies were based on functional magnetic resonance imaging, transcranial magnetic stimulation, and positron emission tomography scan [13-15]. In the majority of lower-income countries, these neuroimaging facilities are only limited to a few tertiary hospitals; thus, it is inconvenient to adopt such practice assessments in a contextual setting. Therefore, other methods of assessments must be sought, and the use of cheaper and conveniently accessible neurobiological signals need to be further investigated for this specific purpose.

Capturing neurobiological signals using electroencephalogram (EEG) that is cheaper and readily available would be more contextually plausible for identifying neuroplasticity changes with stroke rehabilitation. Various studies have been conducted for detecting neuroplasticity effect based on EEG signal analysis in stroke rehabilitation, but these studies are confined in investigating the direct effect of rehabilitation therapy on the neuroplasticity [16-20]. Further investigation on the relationship between neuroplasticity and rehabilitation therapy intensity after stroke were not evaluated in detail. Analyzing parameters of these neurobiological signals after rehabilitation therapy would provide in-depth knowledge on the relationship between neuroplasticity after stroke and therapy intensity.

The activation of neurobiological signals from the affected stroke areas should demonstrate incremental changes with time if rehabilitation therapy is conducted more frequently. It is hypothesized that the neuroplasticity changes occurring with rehabilitation can be objectively measured through the association between analyzed incremental changes derived from the EEG signals and the improved functional outcome measure scores. Based on this hypothesis, the dissimilarity in the associations observed with different rehabilitation frequency may demonstrate a dose-response relationship. The purpose of this study is to identify and determine the changes in the neurobiological signals from the EEG, to correlate these with the improved functional outcome measure scores after rehabilitation as an objective measurement of neuroplasticity, and to compare the associations observed in different therapy frequency for gait rehabilitation among subacute stroke individuals. These objectives are aimed through an interventional, randomized, single-blinded, controlled study.

## Methods

### Study Type, Blinding, Design, Randomization, Recruitment, and Intervention

The study will be an interventional, randomized, single-blinded, controlled study conducted at an outpatient rehabilitation setting among adult individuals with moderate to severe stroke in the subacute phase. Participants for the study will be recruited at a rehabilitation medicine clinic of a major tertiary hospital in the capital city of Sabah from the period of November 1, 2021, to October 31, 2022. Recruited participants and the in-charge physiotherapist will know the frequency of stroke rehabilitation intervention that each participant would be receiving. However, a separate neurophysiological technologist, EEG signals analyst, and therapist are to be assigned for measuring the outcome measures and analyzing the data without prior knowledge on the type of intervention that each participant will be subjected to.

Informed and recruited participants will be randomized into two groups: one group to receive a high frequency gait training (called the intervention group [IG]) and another group to receive the standard routine, low frequency gait training (called the control group [CG]), in a ratio of 1:1. The expected number of participants for each group is 18 (refer to the Sample Size section). The randomization plan for assigning treatment to each participant will be generated through online randomization software.

**Figure 1:**
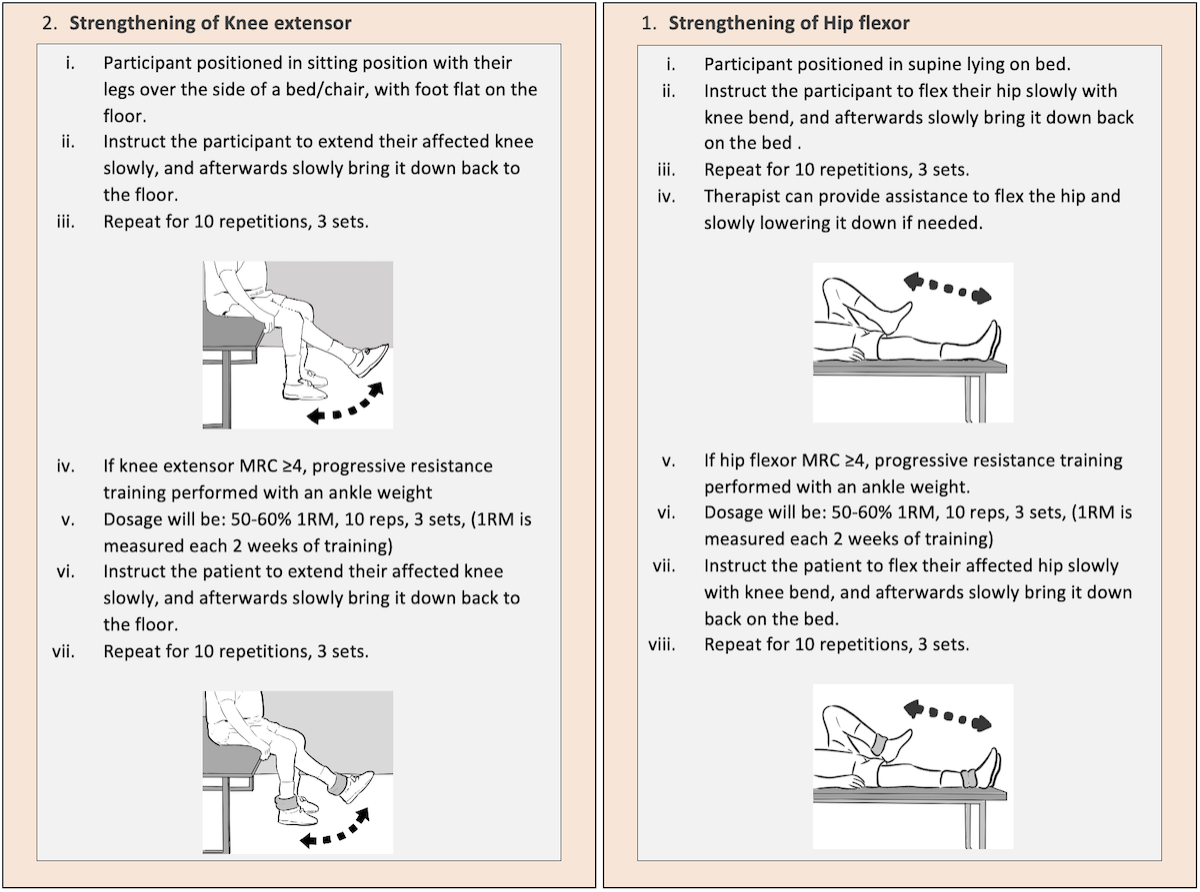
Strengthening exercise for hip flexor and knee extensor.

**Figure 2:**
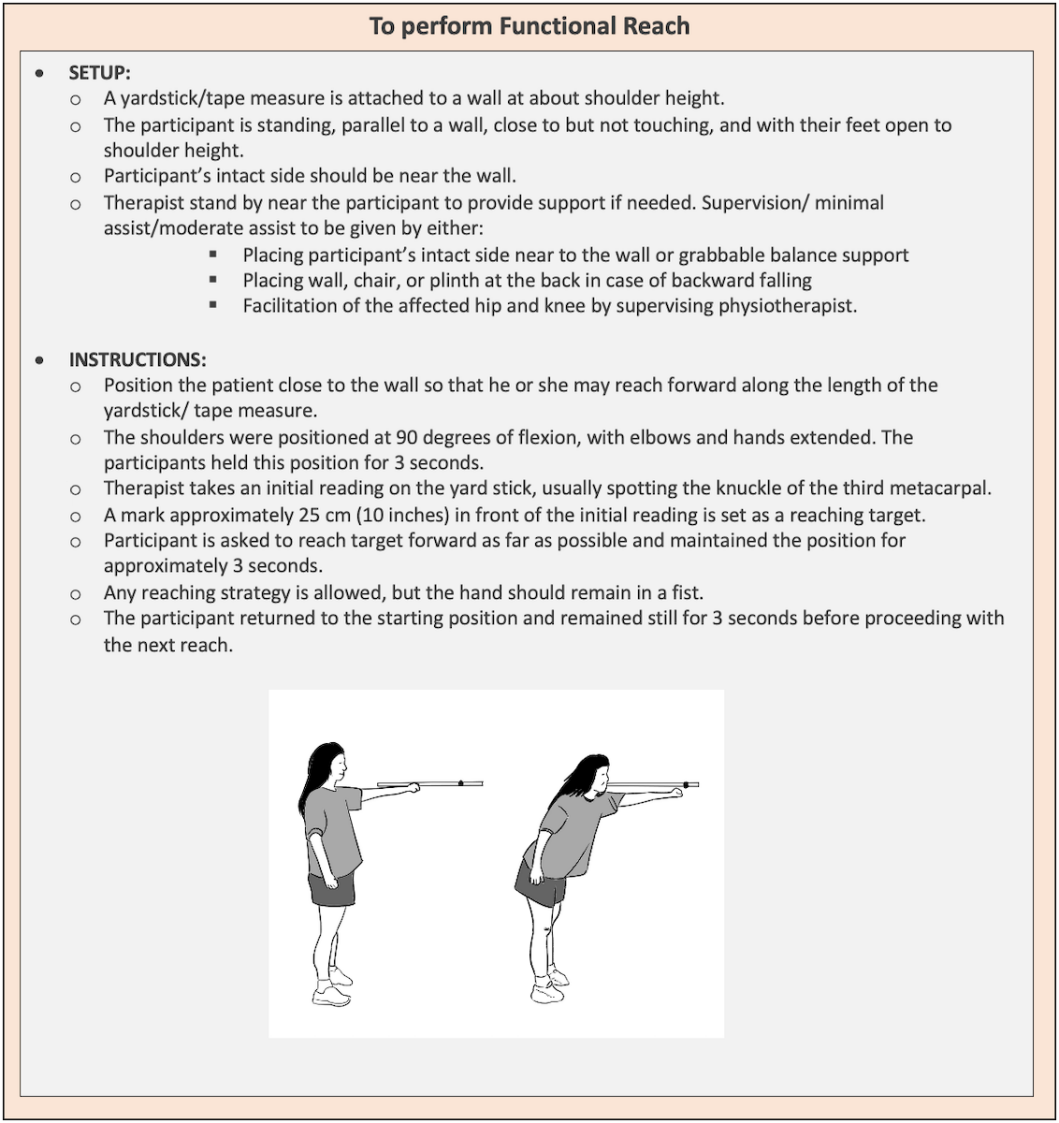
Functional reach for balance training.

**Figure 3:**
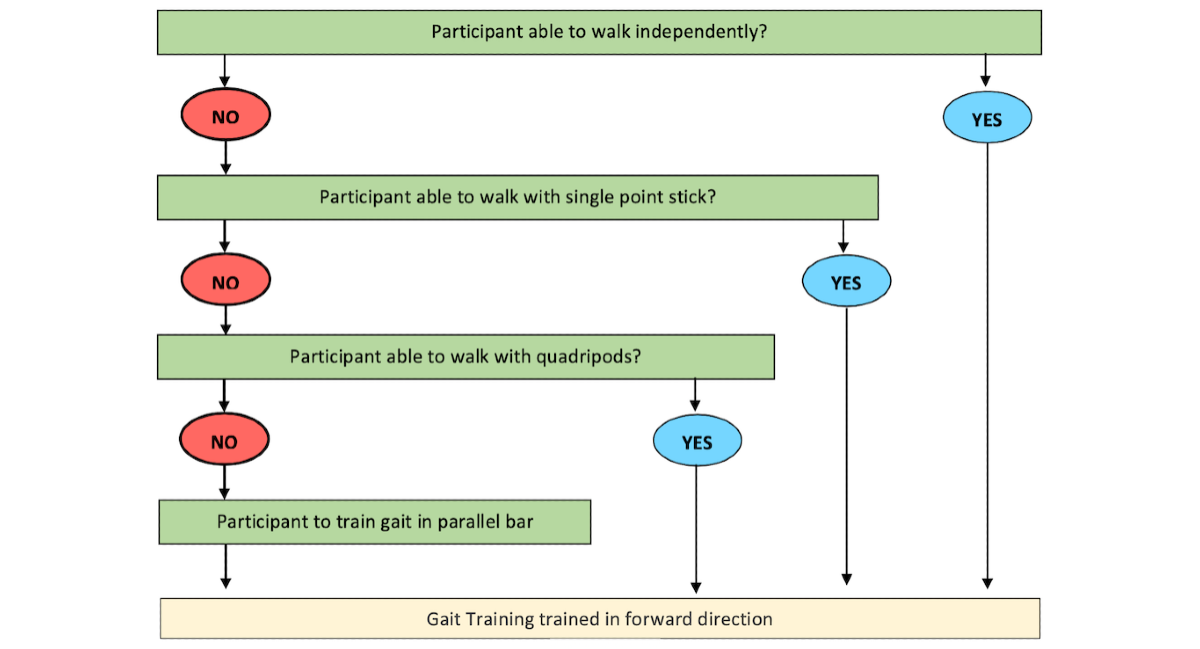
Flowchart on selecting mode of gait training.

Each participant in the IG will have a therapy session three times a week, while a participant in the CG will have a therapy session once a week. Both groups will have to attend the session for a total of 12 consecutive weeks. Each session will last for approximately 40 minutes with a rate of perceived exertion between 3 to 5 minutes as the intensity threshold. The following training will be included: strengthening of hip flexors and knee extensors for approximately 5 minutes (Figure 1); balance training using a functional reach activity for approximately 10 minutes (Figure 2); gait training for approximately 20 minutes and to be conducted with or without walking aids and ankle-foot orthosis, depending on the stability, balance, and confidence level of the participants (Figure 3); and cooling down for approximately 5 minutes.

### Sampling Plan, Data Collection Procedures, and Sample Size

The heterogeneity of stroke might impose difficulty for generalization of the outcomes, hence the need to impart strict eligibility criteria for the study. Inclusion criteria are adult patients older than 18 years, unilateral stroke, ischemic stroke, moderate to severe stroke severity presentation based on admission NIHSS (National Institute of Health Stroke Scale) score of 16 to 24, and displaying hip flexor and knee extensor muscle strength of MRC grade 3 or above during the subacute phase (at 3-6 months after stroke) before they are randomized for intervention. Exclusion criteria to be considered include recurrent stroke, lactating mothers, pregnant women, brain stem stroke, bilateral limbs (either upper or lower) weakness, reduced cognitive function based on a Mini Mental State Screening score of 18 and less, significant dysphasia, underwent poststroke craniectomy, and presence of severe spasticity or contracture.

One physiotherapist in charge is assigned to monitor participants’ therapy, and another will be assigned to ensure blind assessments of the functional outcome measures. The end point of the study for each participant will be achieved when the final assessment is completed at 12 weeks after the intervention has ended or if adverse events are occurring during the study period.

Based on several studies [16-20], the studied population ranges from 10 to 40. A randomized controlled study by Calabrò et al [17] had 20 patients in each arm. At the same time, based on expert consultation from the neurologist at the study site, roughly 3 to 4 new stroke cases that meet the eligibility criteria will be admitted. Considering that the study is focusing on the subacute stroke population, with limited cases to be recruited, the proposed study sample is finalized to 30 participants in each arm.

### Variables and Outcome Measures

Two major independent variables for evaluation are patients’ demographic and stroke-related clinical information. Patient demographics shall include age, gender, and ethnicity. Stroke-related clinical information will incorporate the admission NIHSS score for classifying stroke severity, duration after stroke, and type of stroke.

The primary outcomes for assessment are brain wave frequencies and functional outcome measures. Brain wave frequencies from EEG signals will be conducted through resting-state EEG recordings at preintervention (R0) and postintervention (R1; within 2-4 weeks before the first and 2-4 weeks after the final therapy session) with the participant in a comfortable supine position. The participant will have to keep the eyes closed while awake and relaxed for 3 minutes during the EEG recording. The signals acquisition will require the use of a cap with 32 scalp monopolar electrodes placed according to the International 10/20 system.

Functional outcome measures based on the 6-Minute Walk Test (6MWT), Motor Assessment Scale (MAS), Berg Balance Scale (BBS), and Modified Barthel Index (MBI) will be assessed at preintervention (R0) and postintervention (R1; before the first and after the final therapy session). The 6MWT assesses endurance level through distance covered as a functional walking test [21]. The MAS is a performance-based scale for assessing everyday motor function in patients with stroke [22]. The BBS measures balance impairment, and its usability in poststroke assessment has been validated [23]. The MBI evaluates functional performances (feeding, bathing, grooming, dressing, bowel control, bladder control, toileting, chair transfer, ambulation, and stair climbing) with a maximum score of 100 [24].

### Statistical Analysis and Modeling

Descriptive analysis will be tabulated for demographics, clinical information, and functional measures. Descriptive data will be expressed as means and SDs unless otherwise stated. SPSS version 22 (IBM Corp) will be used for data analysis. One-way analysis of variance (ANOVA) will be used for analysis of normally distributed variables. Kruskal-Wallis ANOVA will be used for nonnormally distributed data. Categorical data will be analyzed using chi-square or Fisher exact test. A value of *P*<.05 is considered statistically significant. The data on the EEG signals will be displayed with graphs whenever appropriate.

The critical analytic comparisons will be as follows:

- Changes of functional outcome measures based on the 6MWT, MAS, BBS, and MBI scores at R0 and R1 in the CG and IG groups
- Changes of brain wave frequencies derived from EEG signals at R0 and R1 in the CG and IG groups
- Admission NIHSS score and admission lower extremity motor score based on NIHSS in the CG and IG groups

### Quantitative EEG Indices Analysis

The brain wave frequencies derived from EEG signals are primarily captured for quantitative EEG (qEEG) indices analysis. The four main types of brain wave frequencies are delta (1-4 Hz), theta (4.1-8 Hz), alpha (8.1-12.5 Hz), and beta (12.6-30 Hz). In many established neurophysiological studies, two commonly used qEEG indices are delta to alpha ratio (DAR) and delta-plus-theta to alpha-plus-beta ratio (DTABR). DAR is proven to have the potential for improving the assessment of cerebrovascular pathology and found to be associated with NIHSS score after 30 days of stroke for prognostication [25]. On the other hand, NIHSS scores were not substantially linked with qEEG assessments of theta or beta waves. DTABR is considered as one of the best qEEG indices and demonstrated to be more superior than the ASPECTS measure for predicting poststroke outcome at discharge and up to 12 months after the event [26].

Data analysis for EEG signals will be performed offline in MATLAB (MathWorks) using the EEGLAB toolbox. This process will start by exporting the EEG raw data and saving it in the form of a Mat file for further evaluation. The EEG data is planned to be resampled at 500 Hz, then undergo several filters (bandpass, lowpass, and highpass) before being divided into consecutive nonoverlapping epochs, and will be mean detrended. The bad channels and segments containing gross artifacts that can be identified by visual inspection will be excluded. Next, independence component analysis will be used to eliminate other artifacts such as loss of electrode connections, ocular artifacts, and muscle artifacts. To extract the spectral power from the EEG data, each resultant component will undergo the time series, the topographic distribution of signal amplitudes, frequency spectra, and frequency loading. The spectral analysis will be done using a specific epoch of discrete fast Fourier transform to obtain and monitor certain frequency resolution.

### Association Analysis

The changes observed in the DAR and DTABR will be further analyzed for association with the changes in 6MWT, MAS, BBS, and MBI for both the CG and IG. It is expected that both associations, based on correlation analyses, would be positive in both groups. However, the *r* value derived from the correlation is expected to be higher in the CG, grounded on the hypothetical assumption that the neuroplasticity changes would be enhanced in higher frequency training.

### Ethics Consideration

This study has received the ethics approval from the National Medical Research Register of Malaysia, which is the formal and statutory body that governed all medical-related studies in this country, with ID no NMRR-19-3840-51591 (IIR).

## Results

Data analysis will be conducted after interventions are completed for all recruited patients. Of 24 eligible stroke patients attending the study site, we have recruited 18 participants so far and expect to publish our results in early 2023. The remaining 6 patients had logistic issues; hence, they were not able to participate in the study procedure.

## Discussion

The findings from this study would objectively demonstrate the enhanced neuroplasticity changes occurring with a higher frequency of rehabilitation training. The significance of these findings explain two major concepts in neuroplasticity post stroke: (1) the enhanced neuroplasticity implies that the stroke recovery with rehabilitation is exponential, likely due to a larger recruitment of synaptogenesis, and (2) a dose-response relationship for poststroke recovery. The dose refers to the frequency of therapy session, and the response reflects the neuroplasticity changes. Demonstrating this effect for the first time would permit a better understanding on the extent of stroke recovery so that the rehabilitation regime delivered is guided based on a more objective manner, rather than a blanket approach for all.

## Abbreviations

ANOVA: analysis of variance
BBS: Berg Balance Scale
CG: control group
DAR: delta to alpha ratio
DTABR: delta-plus-theta to alpha-plus-beta ratio
EEG: electroencephalogram
IG: intervention group
MAS: Motor Assessment Scale
MBI: Modified Barthel Index
NIHSS: National Institute of Health Stroke Scale
qEEG: quantitative electroencephalogram
6MWT: 6-Minute Walk Test

## References

[1] Singh R, Chen S, Ganesh A, Hill MD (2018). Long-term neurological, vascular, and mortality outcomes after stroke. Int J Stroke.

[2] Ting WK, Fadul FA, Fecteau S, Ethier C (2021). Neurostimulation for stroke rehabilitation. Front Neurosci.

[3] Yusoff AF, Mustafa AN, Kaur GK, Omar MA, Vos T, Rao VPC, Begg S (2005). Malaysian burden of disease and injury study.

[4] Bunketorp-Käll L, Lundgren-Nilsson Å, Samuelsson H, Pekny T, Blomvé K, Pekna M, Pekny M, Blomstrand C, Nilsson M (2017). Long-term improvements after multimodal rehabilitation in late phase after stroke: a randomized controlled trial. Stroke.

[5] Burridge J, Lee A, Turk R, Stokes M, Whitall J, Vaidyanathan R, Clatworthy P, Hughes A, Meagher C, Franco E, Yardley L (2017). Telehealth, wearable sensors, and the internet: will they improve stroke outcomes through increased intensity of therapy, motivation, and adherence to rehabilitation programs?. J Neurol Phys Ther.

[6] Bird M, Miller T, Connell LA, Eng JJ (2019). Moving stroke rehabilitation evidence into practice: a systematic review of randomized controlled trials. Clin Rehabil.

[7] Platz T (2019). Evidence-based guidelines and clinical pathways in stroke rehabilitation-an international perspective. Front Neurol.

[8] Alia C, Spalletti C, Lai S, Panarese A, Lamola G, Bertolucci F, Vallone F, Di Garbo A, Chisari C, Micera S, Caleo M (2017). Neuroplastic changes following brain ischemia and their contribution to stroke recovery: novel approaches in neurorehabilitation. Front Cell Neurosci.

[9] Du J, Yang F, Hu J, Hu J, Xu Q, Cong N, Zhang Q, Liu L, Mantini D, Zhang Z, Lu G, Liu X (2019). Effects of high- and low-frequency repetitive transcranial magnetic stimulation on motor recovery in early stroke patients: Evidence from a randomized controlled trial with clinical, neurophysiological and functional imaging assessments. Neuroimage Clin.

[10] Chaturvedi P, Singh AK, Kulshreshtha D, Maurya PK, Thacker AK (2018). Proprioceptive neuromuscular facilitation (PNF) vs. task specific training in acute stroke: the effects on neuroplasticity. MOJ Anat Physiol.

[11] Pin-Barre C, Hugues N, Constans A, Berton E, Pellegrino C, Laurin J (2021). Effects of different high-intensity interval training regimens on endurance and neuroplasticity after cerebral ischemia. Stroke.

[12] Belagaje SR (2017). Stroke rehabilitation. Continuum Lifelong Learning Neurol.

[13] Abdullahi A, Truijen S, Saeys W (2020). Neurobiology of recovery of motor function after stroke: the central nervous system biomarker effects of constraint-induced movement therapy. Neural Plast.

[14] Auriat AM, Neva JL, Peters S, Ferris JK, Boyd LA (2015). A review of transcranial magnetic stimulation and multimodal neuroimaging to characterize post-stroke neuroplasticity. Front Neurol.

[15] Grefkes C, Fink GR (2020). Recovery from stroke: current concepts and future perspectives. Neurol Res Pract.

[16] Ding L, Wang X, Chen S, Wang H, Tian J, Rong J, Shao P, Tong S, Guo X, Jia J (2019). Camera-based mirror visual input for priming promotes motor recovery, daily function, and brain network segregation in subacute stroke patients. Neurorehabil Neural Repair.

[17] Calabrò RS, Naro A, Russo M, Bramanti P, Carioti L, Balletta T, Buda A, Manuli A, Filoni S, Bramanti A (2018). Shaping neuroplasticity by using powered exoskeletons in patients with stroke: a randomized clinical trial. J Neuroeng Rehabil.

[18] Pan LH, Yang W, Kao C, Tsai M, Wei S, Fregni F, Chen VC, Chou L (2018). Effects of 8-week sensory electrical stimulation combined with motor training on EEG-EMG coherence and motor function in individuals with stroke. Sci Rep.

[19] Trujillo P, Mastropietro A, Scano A, Chiavenna A, Mrakic-Sposta S, Caimmi M, Molteni F, Rizzo G (2017). Quantitative EEG for predicting upper limb motor recovery in chronic stroke robot-assisted rehabilitation. IEEE Trans Neural Syst Rehabil Eng.

[20] Ingemanson ML, Rowe JR, Chan V, Wolbrecht ET, Reinkensmeyer DJ, Cramer SC (2019). Somatosensory system integrity explains differences in treatment response after stroke. Neurology.

[21] Kosak M, Smith T (2005). Comparison of the 2-, 6-, and 12-minute walk tests in patients with stroke. J Rehabil Res Dev.

[22] Carr J, Shepherd RB, Nordholm L, Lynne D (1985). Investigation of a new motor assessment scale for stroke patients. Phys Ther.

[23] Blum L, Korner-Bitensky N (2008). Usefulness of the Berg Balance Scale in stroke rehabilitation: a systematic review. Phys Ther.

[24] Collin C, Wade DT, Davies S, Horne V (1988). The Barthel ADL Index: a reliability study. Int Disabil Stud.

[25] Finnigan SP, Walsh M, Rose SE, Chalk JB (2007). Quantitative EEG indices of sub-acute ischaemic stroke correlate with clinical outcomes. Clin Neurophysiol.

[26] Bentes C, Peralta AR, Viana P, Martins H, Morgado C, Casimiro C, Franco AC, Fonseca AC, Geraldes R, Canhão P, Pinho E Melo T, Paiva T, Ferro JM (2018). Quantitative EEG and functional outcome following acute ischemic stroke. Clin Neurophysiol.

